# A Combined Experimental and Theoretical Study on the Immunoassay of Human Immunoglobulin Using a Quartz Crystal Microbalance

**DOI:** 10.3390/s101211498

**Published:** 2010-12-15

**Authors:** Po-Jen Liao, Jeng-Shian Chang, Sheng D. Chao, Hung-Chi Chang, Kuan-Rong Huang, Kuang-Chong Wu, Tzong-Shyan Wung

**Affiliations:** Institute of Applied Mechanics, National Taiwan University, Taipei 106, Taiwan; E-Mails: d94543005@ntu.edu.tw (K.R.H.); wukc@iam.ntu.edu.tw (K.C.W.); wung@iam.ntu.edu.tw (T.S.W)

**Keywords:** biosensor, Quartz Crystal Microbalance, Finite Element Method (FEM), basic kinetic analysis, human IgG1

## Abstract

We investigate a immunoassay biosensor that employs a Quartz Crystal Microbalance (QCM) to detect the specific binding reaction of the (Human IgG1)-(Anti-Human IgG1) protein pair under physiological conditions. In addition to experiments, a three dimensional time domain finite element method (FEM) was used to perform simulations for the biomolecular binding reaction in microfluidic channels. In particular, we discuss the unsteady convective diffusion in the transportation tube, which conveys the buffer solution containing the analyte molecules into the micro-channel where the QCM sensor lies. It is found that the distribution of the analyte concentration in the tube is strongly affected by the flow field, yielding large discrepancies between the simulations and experimental results. Our analysis shows that the conventional assumption of the analyte concentration in the inlet of the micro-channel being uniform and constant in time is inadequate. In addition, we also show that the commonly used procedure in kinetic analysis for estimating binding rate constants from the experimental data would underestimate these rate constants due to neglected diffusion processes from the inlet to the reaction surface. A calibration procedure is proposed to supplement the basic kinetic analysis, thus yielding better consistency with experiments.

## 1. Introduction

Efficient, accurate, and real-time monitoring of chronic diseases becomes more and more important for an aging society. Biosensors provide a quick and convenient technology for real-time surveillance in health-care. Biosensors use a receptor molecule (the ligand) fixed on the substrate as the bio-recognition layer. When the specific target molecules (the analyte) carried by the buffer solution flow over the reaction surface of a biosensor, a specific binding reaction occurs between the analyte molecules and the immobilized ligand molecules. A variety of physical mechanisms have been used in the transducer to record the specific binding and the subsequent real-time examination takes place by amplifying these signals [1]. With its superior characteristics of timely reaction and high sensitivity, the Quartz Crystal Microbalance (QCM) has recently become a commonly used biosensor. The QCM uses the indirect piezoelectric effect as a way of energy transformation to timely record the resonance frequency shifts with a tiny mass loading. In 1959, Sauerbrey [2] derived an equation (called Sauerbrey equation) to relate the change of the resonance frequency shift to the change of loaded mass on the crystal surface; namely, $\Delta f=-2f_{0}\Delta M/\text{A}\sqrt{\mu_{Q}\rho_{Q}}$, where Δ*f* is the frequency shift, Δ*M* is the change of the load mass, *f*_0_ is the oscillating frequency of the quartz without loaded mass, *μ_Q_* is the elastic modulus of the quartz, *ρ_Q_* is the density of the quartz and *A* is the area of the electrode. Initially, QCM was applied as a gas-sensing device [3]; nowadays, it is widely used in research on bioimmune tests [4,5].

In this study, we use a Quartz Crystal Microbalance for detecting and tracking the specific binding reaction between Human IgG1 and Anti-Human IgG1. The mass change due to the formation of the (Human IgG1)-(Anti-Human IgG1) complex was recorded as the frequency shift *versus* time, which reflects the time evolution of the analyte concentration, the observable of most concern in a clinical diagnosis. Following the conventional procedure, a direct kinetic analysis based on the experimental data can be employed to estimate the binding rate constants, which are then used in the follow-up numerical studies of the binding reaction. We performed three dimensional finite element simulations of the binding reaction and compared our simulation results with the experimental data. Surprisingly, large discrepancies were found between the predicted and the experimental results. We indentified two major issues in the conventional analysis that could cause such inaccurate predictions. The first is the assumption of uniform and time-independent profile of the analyte concentration at the inlet of the micro-channel and the second is the inaccurate estimation of the binding rate constants.

In the experiments, we used a transportation tube conveying the analyte solution to the micro-channel. The cross-sectional concentration profile of the analyte at the end of the transportation tube, which is also the inlet to the micro-channel, is usually assumed to be uniform and time-independent in the simulations. However, when the transportation tube is long, the deviation of the analyte concentration profile from uniformity across the tube section and time-independence is large [6–8]. In this work, we will show that the effect of such non-uniformity and time-dependence of the analyte concentration profile is important for analyzing the binding behavior and should be taken into account during the simulation.

Binding rate constants are usually estimated directly from a basic kinetic analysis of the experimental data under the assumption [9] that the concentration of the analyte near the surface of the biosensor is the same as that in the bulk of the fluid. This assumption in fact leads only to an “apparent” binding rate constant which may significantly differ from the “true” one because the diffusion processes from the inlet to the reaction surface cannot be neglected in a real situation. This can be cross-checked by an inverse calculation of the “apparent” binding rate constants from the simulated binding reaction curves, where the “true” rate constants are assumed to be known *a priori*. We show that the “apparent” rate constants underestimate the “true” ones. Therefore, an effective calibration procedure is proposed to amend the estimation of binding rate constants. The calibrated binding rate constants are then deemed to be the “true” binding rate constants and used to do further numerical simulations. Using this procedure, numerical predictions self-consistent with the experimental data are then found.

This paper is organized as follows. In Section 2 we describe the detailed experimental procedures and the results. The governing equations used in the simulations are presented in Section 3. The detailed theoretical analysis is presented in Section 4. We compare and discuss the experimental and theoretical results in Section 5 and a brief summary is provided in Section 6.

## 2. Experiment

### 2.1. Materials and Methods

**Figure 1.**
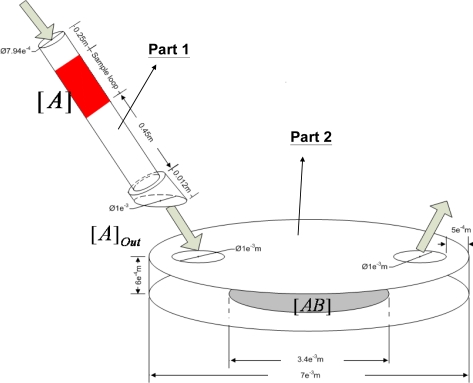
Sketch of the 3D model of the QCM device. Part 1 is the transportation tube conveying the analyte solution into the micro-channel. Part 2 is the micro-channel with the reaction surface.

Figure 1 shows a schematic sketch of the Quartz Crystal Microbalance (Affinity-Sensor New Technology, Taiwan) used in our experiments.

This QCM system consists of a flow-injection system and a QCM chip. The QCM chip is a 9-MHz quartz crystal placed as a sandwich between two gold electrodes and driven by an oscillator circuit to produce the oscillating frequency. The upper gold electrode is also used as the reaction surface. The flow-injection system uses a peristaltic pump to supply the QCM with a steady and continuous amount of phosphate-buffered saline (PBS, pH 7.2, 22 °C) at a flow rate of 50 μL/min. The designated supplement of the analyte solution is continuously injected and stored in the sample loop, and placed between the buffer solutions via the injection valve, as shown in Figure 1. Then, the analyte solution can follow with the running buffer solution into the micro-channel where the QCM chip sits on the bottom side.

### 2.2. Experiment Procedure

In these immunoassay experiments, we used the QCM to monitor the real-time specific binding reaction of the immobilized ligand Human IgG1 (I5154, Sigma) and the Anti-Human IgG1 (I2513, Sigma) analyte. We considered three groups according to the total volume of Anti-Human IgG1 solution supplied during the individual experiments: 800, 500 and 100 μL, respectively. Each group also included four concentrations of Anti-Human IgG1 solution: 50, 25, 10 and 5 μg/mL. Before the experiment started, a self-assembled monolayer (SAM) of the linker molecules was formed on the QCM chip by immersing their upper gold electrode surface in 16-mercaptohexadecanoic acid (MHDA). The covalent bonded linkers served to efficiently immobilize the ligand bio-molecules on the reaction surface. There were four steps in the experiment process. First, a 2.5% glutaraldehyde solution in PBS was added into the QCM to activate the linker on the reaction surface. Following the activation a running PBS solution is used to rinse away the excess glutaraldehyde. The second step is to inject the ligand solution (Human IgG1 50 μg/mL in PBS) into the QCM to bind covalently with the linkers to form the immobilized layer on the reaction surface. Again the excess immobilization ligands were rinsed away with a running PBS solution. The third step is to add a glycine solution (1 M in PBS) to block the unbound linkers to prevent the occurrence of non-specific binding. After the blocking, the running PBS solution again rinses away the excess glycine. In the last step, the designated supplement (800, 500 or 100 μL) of Anti-Human IgG1 solution of various concentrations (50, 25, 10 or 5 μg/mL) was added into the QCM to react specifically with the immobilized Human IgG1.

### 2.3. Experimental Results

**Figure 2.**
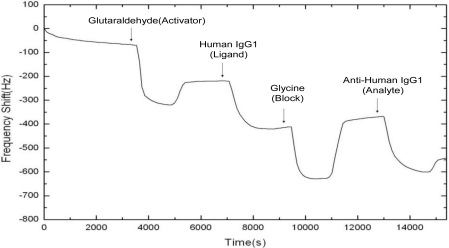
A typical result of the QCM experiment. There are four steps in the experimental process, as described in the text.

**Figure 3.**
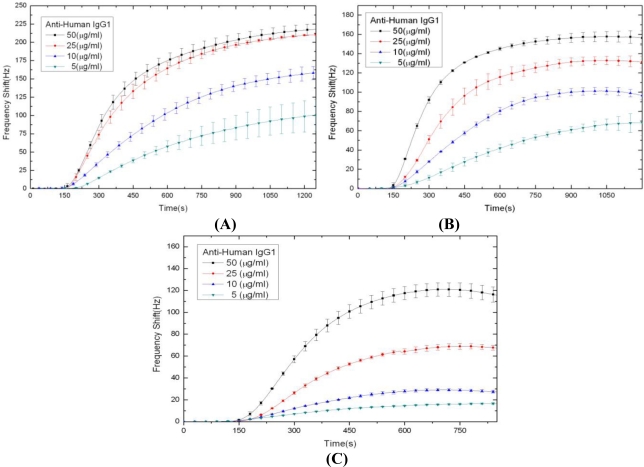
The (Human IgG1)-(Anti-Human IgG1) protein pair binding curves. The supplemented volume of the Anti-Human IgG1 solution is: **(A)** 800 μL, **(B)** 500 μL and **(C)** 100 μL. For each case there are four concentrations of Anti-Human IgG1 solution (50, 25, 10 and 5 μg/mL). The error bar at each time point is marked according to 4∼5 replicates of the experimental raw data; namely picking the largest positive (negative) deviation value as our upper (lower) bound to the average value.

Figure 2 shows a typical result of the frequency shift *versus* time in a QCM experiment. Notice that for convenience and better visibility, we present only the magnitude of the frequency shift (drop) in the rest figures of this paper. Figures 3(A–C) present all of our experimental results of the binding curves for the Human IgG-Anti-Human IgG protein pair for the total volumes of Anti-Human IgG1 solution supplied during the individual experiments (800, 500 and 100 μL, respectively). Each subfigure in Figure 3 contains four curves corresponding to the four different concentrations of the Anti-Human IgG1 solution: 50, 25, 10 and 5 μg/mL, respectively. Comparing the curves presented in Figure 3, it is observed that the behavior of binding reaction depends not only on the concentration of the Anti-Human IgG1 solution but also on the amount of Anti-Human IgG1 solution supplied.

## 3. Simulation

In this work the 3D simulation on the immunoassay in the QCM device is performed using the finite element analysis software, COMSOL Multiphysics [10], to simulate the experiments done in the above section for the (Human IgG1)-(Anti-Human IgG1) binding interactions. The equations governing the flow field, the concentration field and the biochemical reaction are described in this section. Detailed geometry, flow properties, binding constants and other conditions that are required for simulation are described in Section 4.

### 3.1. The Flow Field

In this work it is assumed that the fluid is incompressible so that:

(1) $$\frac{\partial u}{\partial x}+\frac{\partial v}{\partial y}+\frac{\partial w}{\partial z}=0$$

where *u*, *v* and *w* are the *x*, *y* and *z* velocity components, respectively. The equations of motion are:

(2) $$\begin{array}{l} \rho \frac{\partial u}{\partial t}+\rho \left(u\frac{\partial u}{\partial x}+v\frac{\partial u}{\partial y}+w\frac{\partial u}{\partial z}\right)-\eta \nabla^{2}u+\frac{\partial p}{\partial x}=0\\ \rho \frac{\partial v}{\partial t}+\rho \left(u\frac{\partial v}{\partial x}+v\frac{\partial v}{\partial y}+w\frac{\partial v}{\partial z}\right)-\eta \nabla^{2}v+\frac{\partial p}{\partial y}=0\\ \rho \frac{\partial w}{\partial t}+\rho \left(u\frac{\partial w}{\partial x}+v\frac{\partial w}{\partial y}+w\frac{\partial w}{\partial z}\right)-\eta \nabla^{2}w+\frac{\partial p}{\partial z}=0 \end{array}$$

where *η* is the dynamic viscosity of the fluid and *p* is the pressure. In this work it is assumed that the density *ρ* and *η* viscosity of the modeled incompressible fluid are constant independent of temperature and concentration.

### 3.2. The Concentration Field

Transport of the analyte to and from the reaction surface is assumed to be described by Fick’s second law with convective terms:

(3) $$\frac{\partial [A]}{\partial t}+u\frac{\partial [A]}{\partial x}+v\frac{\partial [A]}{\partial y}+w\frac{\partial [A]}{\partial z}=D(\frac{\partial^{2}[A]}{\partial x^{2}}+\frac{\partial^{2}[A]}{\partial y^{2}}+\frac{\partial^{2}[A]}{\partial z^{2}})$$

where [*A*] is the concentration of the analyte and *D* is the diffusion coefficient of the analyte.

### 3.3. The Reaction Surface

The reaction between immobilized ligand and analyte is assumed to follow the first order Langmuir adsorption model [11,12]. During the reaction, the reaction complex [*AB*] increases as a function of time according to the reaction rate:

(4) $$\frac{\partial [AB]}{\partial t}=k_{a}{\left[A\right]}_{\text{surface}}([B_{0}]-[AB])-k_{d}[AB]$$

where [*A*]*_surface_* is the concentration of the analyte at the reaction surface by mass-transport, [B_0_] is the surface concentration of the ligand on the reaction surface, and [*AB*] is the surface concentration of the reaction complex.

## 4. Simulation Detail and Kinetic Analysis

As shown in Figure 1, our QCM device consists of a Part 1 and a Part 2. Part 1 is the tube loop for storing and transporting the sample solutions into the micro-channel. The lengths of the sample loop are 1.62, 1.10 and 0.20 m, corresponding to 800, 500 and 100 μL analyte supplements, respectively. Part 2 is the micro-channel where the QCM sensor is placed. The reaction surface is located at the bottom of the micro-channel. The dimensions of the 3D model are depicted in Figure 1. The analyte solution together with the buffer solution flows through the tube and passes through the left inlet to the right outlet of the micro-channel. The flow field in the tube is a fully developed laminar flow. The concentration profile of the analyte at the tube outlet (that is, inlet of the micro-channel) is strongly affected by the flow field in the tube. It is shown below that this concentration profile at the tube outlet is in fact not uniform and time-independent. This will in turn affect significantly the behavior of the binding reaction occurred on the QCM. Therefore, we shall first discuss the distribution of the analyte concentration at the tube outlet in Part 1, and then use the results obtained in Part 1 to simulate the reaction curves of the ligand and analyte in Part 2.

### 4.1. The Flow Field

The value of dynamic viscosity *η* is set as that of water, 6.96 × 10^−3^ Pa·s. Since the flows in the tube and micro-channel are in low Reynolds number condition, it is assumed that the flow is laminar. The average velocity of the parabolic profile is set to *u* = 1.68× 10^−3^ m/s in the tube according to the experiment condition. The pressure at the micro-channel outlet is *p* = 0.

### 4.2. The Concentration Field

The diffusion coefficient of Anti-Human IgG1 is 5 × 10^−11^ m^2^/s [13]. The initial concentrations of the analyte in the tube are chosen as [*A*] = 3.125 × 10^−4^ mol/m^3^ (50 μg/mL), 1.5625 × 10^−4^ mol/m^3^ (25 μg/mL), 6.25 × 10^−4^ mol/m^3^ (10 μg/mL) and 3.125 × 10^−5^ mol/m^3^ (5 μg/mL). The initial surface concentrations [*B*_0_] are assumed as 7.8 × 10^−8^ mol/m^2^ in the model of 800 μL, 5.6 × 10^−8^ mol/m^2^ in the model of 500 μL and 5.2 × 10^−8^ mol/m^2^ in the model of 100 μL from the experimental results. Since [*B*_0_] is not a constant in our experiments, the following normalized equation is used to represent the reaction between ligand and analyte:

(5) $$\frac{1}{\left[B_{0}\right]}\frac{\partial [AB]}{\partial t}={\text{k}}_{\text{a}}{\left[\text{A}\right]}_{\text{surface}}\left(1-\frac{\left[AB\right]}{B_{0}}\right)-k_{d}\frac{\left[AB\right]}{\left[B_{0}\right]}$$

### 4.3. Kinetics of the Specific Binding

The specific recognition of the analytes and immobilized ligands occurs on the reaction surface. The reaction kinetics can be described as a two-step process [14].

Mass-transport process: the analyte is transported by diffusion from the bulk solution toward the reaction surface:
  (6) $${\left[A\right]}_{\text{bulk}}⇆{\left[A\right]}_{\text{surface}}$$
Chemical reaction process: the binding of the protein pair takes place:
  (7) $${\left[A\right]}_{\text{surface}}+[B]\overset{k_{a}}{\underset{k_{d}}{⇄}}[AB]$$
  where [*A*]*_bluk_*, [*A*]*_surface_*, [*B*] and [*AB*] are the concentrations of the analyte in the bulk, the analyte at the reaction surface, the ligand on the reaction surface, and the analyte-ligand complex on the reaction surface, respectively. *k_a_* and *k_d_* are the association and dissociation rate constants, respectively.

Conventionally, the association rate constant *k_a_* and dissociation rate constant *k_d_* of the specific protein pairs can be estimated from the experimental data by a basic kinetic analysis [9]. In this basic kinetic analysis, the mass-transport process is assumed so fast that the surface concentration [*A*]*_surface_* is the same as the bulk concentration[*A*]_bluk_, which is true for QCM working in an air environment. However, such an assumption is not valid in a solution environment since the diffusion of biomolecules, especially for the large molecules like Anti-Human IgG1, is slow in liquids. In addition, the flow speed in the micro-channel is usually also low, which limits the convective transport of the analyte. Therefore, the conventional analysis actually yields the “apparent” association and dissociation rate constants *k′_a_* and *k′_d_*, but not the “true” ones. To remedy this deficiency in the basic kinetic analysis, in the following we propose a modified method, which calibrates the calculation of the conventional kinetic analysis, to recover the “true” reaction rate constants.

#### 4.3.1. Basic Kinetic Analysis and Calculation of Reaction Rate Constants

The “apparent” reaction rate constants *k′_a_* and k′*_d_* are estimated by the method of Karlsson [14] under the assumption [*A*]*_surface_* ≈ [*A*]*_bluk_*, Equation (4) can then be rewritten as:

(8) $$\frac{\partial [AB]}{\partial t}=k_{a}{\left[A\right]}_{\text{surface}}[[B_{0}]-[AB]]-k_{d}[AB]\approx k_{a}^{\prime }{\left[A\right]}_{\text{bulk}}[[B_{0}]-[AB]]-k_{d}^{\prime }[AB]$$

In general, the response *R* is equal to *α*[*AB*], where *α* is a proportional constant. When analyte solution is of very high concentration, all the binding sites of the ligand are occupied and the maximum response *R_max_* is equal to *α*[*B*_0_]. Thus, Equation (8) can be rewritten as:

(9) $$\frac{\partial R}{\partial t}=k_{a}^{\prime }CR_{\text{max}}-(k_{a}^{\prime }C+k_{d}^{\prime })R$$

where the constant *C* is the concentration of the analyte in the bulk, that is [*A*]*_bluk_*. There are usually two methods to retrieve the rate constants.

Method 1:

According to the Equation (9), we can draw a plot of ∂*R*/∂*t versus R* and the plot will have a slope −*k_s_* equal to −(*k′_a_C* + *k′_d_*). Therefore, by calculating ∂*R*/∂*t* from the experimental data of *R versus* time *t*, we can plot the curve of ∂*R*/∂*t versus R*, whose slope is −*k_s_* for a given analyte concentration *C*. Then, the plot of −*k_s_* *versus C* can be drawn to obtain the slope *k′_a_* and the intercept *k′_d_*.

Method 2:

We also can solve *R* from Equation (9) to yield:

(10) $$\text{R}=\frac{k_{a}^{\prime }CR_{\text{max}}}{k_{a}^{\prime }C+k_{d}^{\prime }}(1-e^{-(k_{a}^{\prime }C+k_{d}^{\prime })t})$$

Then, we can obtain the apparent reaction rate constants *k′_a_* and *k′_d_* by curve fitting. Table 1 shows the estimated rate constants using the above two methods. We see that method 1 and method 2 yield similar *k′_a_* and *k′_d_* values.

**Figure 4.**
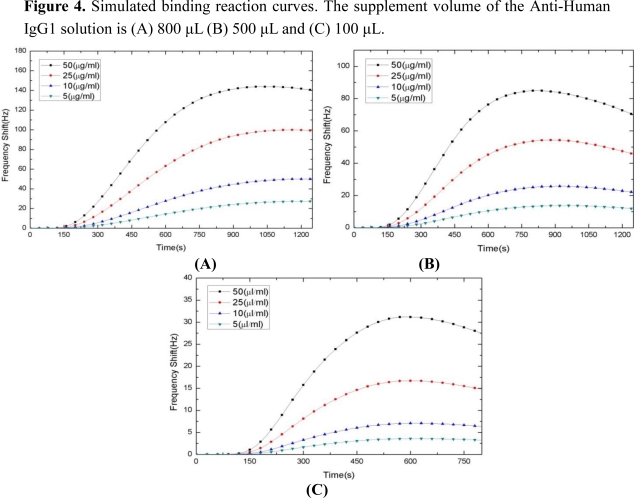
Simulated binding reaction curves. The supplement volume of the Anti-Human IgG1 solution is (A) 800 μL (B) 500 μL and (C) 100 μL.

Next, we use the apparent *k′_a_* and *k′_d_* to perform a three dimensional finite element simulation. The simulation results are presented in Figure 4. By comparing to the experimental data shown in Figure 3, large errors are observed due to the inaccurate estimation of the binding rate constants.

Therefore, we propose below a modified method to calibrate the calculation of the reaction rate constants in the conventional kinetic analysis. The calibrated rate constants will be used in the further 3D simulation to verify their correctness.

#### 4.3.2. Modified Kinetic Analysis

When the reaction reaches saturation, the time variation of the concentration of the analyte-ligand complex vanishes and [*A*]*_surface_* = [*A*]*_bluk_* Equation (8) can then be written as:

(11) $$[B_{0}]={\left[AB\right]}_{\text{sat}}\left(1+\frac{k_{d}/k_{a}}{{\left[A\right]}_{\text{bulk}}}\right)\approx {\left[\tilde{AB}\right]}_{\text{sat}}\;\text{if}{\left[A\right]}_{\text{bulk}}\;\;\gg \;\frac{k_{d}}{k_{a}}$$

In the equation above, if we pick the concentration of the analyte solution to be high (designated [*A*]_bluk_ = [*Ã*]_bluk_) that ${\left[\tilde{A}\right]}_{\text{bulk}}\gg \frac{k_{d}}{k_{a}}$, then Equation (11) yields:

(12) $$[B_{0}]={\left[\tilde{AB}\right]}_{\text{sat}}\;\text{when}{\left[\tilde{A}\right]}_{\text{bulk}}\gg \frac{k_{d}}{k_{a}}$$

where ${\left[\tilde{AB}\right]}_{\text{sat}}$ is the corresponding saturated concentration of the reaction complex when the concentration of the analyte is very high such that ${\left[A\right]}_{\text{bluk}}\gg \frac{k_{d}}{k_{a}}$. Then from Equations (11) and (12), the equilibrium association constant *K_D_* can be computed as:

(13) $$K_{D}=\frac{k_{d}}{k_{a}}={\left[A\right]}_{\text{bulk}}\left(\frac{\left[B_{0}\right]}{{\left[AB\right]}_{\text{sat}}}-1\right)={\left[A\right]}_{\text{bulk}}(\frac{{\left[\tilde{AB}\right]}_{\text{sat}}}{{\left[AB\right]}_{\text{sat}}}-1)$$

The experimental results shown in Figure 3(A) are for the case of 800 μL Anti-Human IgG1 solution, in which the reaction curves for the analyte concentrations [*A*] = 50 μg/mL and 50 μg/mL are saturated, and these two concentrations are deemed as [*Ã*]_bluk_ and [*A*]_bluk_, respectively. So, we use the two corresponding maximum frequency shifts to calculate *K_D_*:

(14) $$K_{D}={\left[A\right]}_{\text{bulk}}\left(\frac{{\left[\tilde{AB}\right]}_{\text{sat}}}{{\left[AB\right]}_{\text{sat}}}-1\right)=1.5625\times 10^{-7}\times \left(\frac{221}{209}-1\right)=8.97\times 10^{-9}(M)$$

Then, we perform the 3D simulation of binding reaction for thirty pairs of trial *k_a_* and *k_d_*, and compute the apparent association rate constant *k′_a_* by the basic kinetic analysis to set up a look-up Table 2.

Then we can use this table and the apparent *k′_a_* calculated from the experimental data (see Table 1) by the basic kinetic analysis to access the “true” *k_a_*, which is roughly 8 × 10^4^ (M^−1^ s^−1^). Since the apparent dissociation rate constant *k′_d_* is very small, direct extrapolation from the basic kinetic analysis would yield large error. Instead, we can compute the “true” *k_d_* by *k_d_* = *k_a_* × *k_D_*, which gives *k_d_* = 7.17 × 10^−4^ (s^−1^). This value is about one fifth of the apparent *k′_d_* (see Table 1).

## 5. Comparative Results

**Figure 5.**
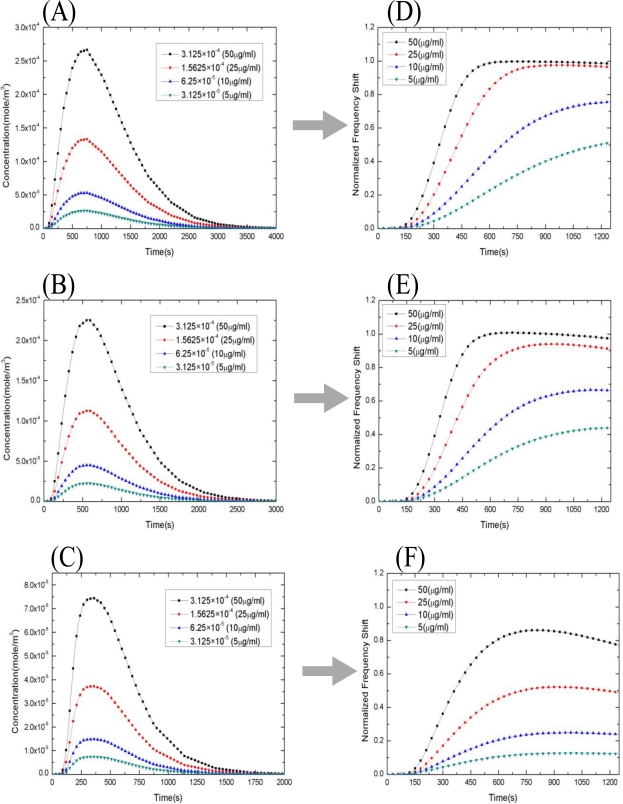
The distribution of the analyte concentration at the outlet of the tube, which is the inlet of the micro-channel, and the corresponding binding reaction curves.

The calibrated *k_a_* and *k_d_* can be found by the modified kinetic analysis in Section 4. Next, we use the calibrated *k_a_* and *k_d_* to simulate the IgG1-Anti-IgG1 binding reaction. First, the analyte concentration profiles at the tube outlet for 800 μL, 500 μL, and 100 μL analyte solutions, respectively, are shown in Figure 5 (A–C). On the right panel of Figure 5, denoted as (D), (E), and (F), are shown the corresponding curves of the binding reaction. Each of the subfigures presents the four curves corresponding to the four different concentrations of Anti-Human IgG1: 50, 25, 10 and 5 μg/mL, respectively. It is clearly seen that the analyte concentration profile in the tube is strongly affected by the laminar flow, and definitely is not uniform and constant. By decreasing the supplement of the analyte, the concentration of the analyte is apparently decayed when length of the tube is fixed. From Figures 5(D–F), we can see that the behavior of the reaction curves depends significantly on the supplement of the analyte. For example, consider the case of high concentration of Anti-Human IgG1 solution, say 50 μg/mL. When the supplement of the Anti-Human IgG1 solution is sufficient, say 800 μL, the reaction time required for saturation of forming Anti-Human IgG1/ Human IgG1 complex (and is the frequency shift) is 600 *s* as shown in Figure 5(D), which falls before the maximum concentration of Anti-Human IgG1 solution, which is about 45 μg/mL (a little less than 50 μg/mL), reached at the inlet of reaction chamber, say 780 *s*, as shown in Figure 5(A). Thus, saturation can be reached and maintained a period of time. In contrast, when the supplement of the Anti-Human IgG1 solution is insufficient, say only 100 μL, Figures 5(C) and 5(F) show that the maximum concentration of Anti-Human IgG1 solution at the inlet of reaction chamber is only 12 μg/mL (much less 50 μg/mL), and the maximum frequency shift is 83% of that obtained when we have 800 μL the supplement of Anti-Human IgG1 solution. In addition, the maximum frequency shift cannot be maintained but starts to fall immediately. As for the case of low concentration of Anti-Human IgG1 solution, say 5 μg/mL, Figures 5(D) and 5(F) show that the maximum frequency shift for 100 μL supplement of the Anti-Human IgG1 solution is only 25% of that for 800 μL supplement of the Anti-Human IgG1 solution.

**Figure 6.**
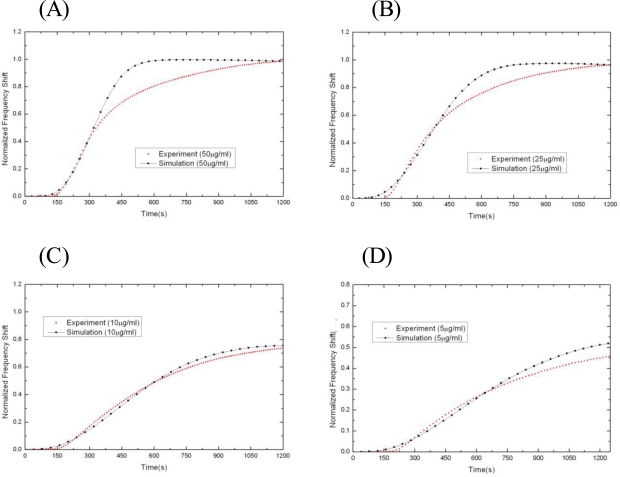
The normalized experimental and simulated binding reaction curves for the 800 μL supplement volume. Here the concentrations of the Anti-Human IgG1 solution are (A) 50 μg/mL, (B) 25 μg/mL, (C) 10 μg/mL, and (D) 5 μg/mL.

We compare the normalized reaction curves of experiment and simulation to verify the accuracy of the simulated model. These results are shown in Figures 6, 7 and 8 for supplements of 800, 500 and 100 μL, respectively (Figures 7 and 8 may be found in the Supplementary material). We see that overall the simulations reproduce the main features of the experimental curves for a wide range of analyte concentrations, including the initial slope of frequency, reaction time to saturation, and maximum frequency shift (reduction), *etc*.

## 6. Conclusions

In this work, immunoassay experiments on Human IgG1 and the Anti-Human IgG1 were monitored with a QCM. In addition, we performed 3D finite element analysis to simulate the (Human IgG1)-(Anti-Human IgG1) binding reactions. We compare the results of the experiments and the simulations to verify the simulation model. Here we summarize our conclusions:

1. The analyte concentration distribution in the tube is strongly affected by the unsteady convective diffusion in the fully developed laminar flow.
2. The assumption of [A]*_surface_* = [A]*_bulk_* is not valid because of the effect of the slower mass transport in a fluid environment than that in the air. The small diffusion coefficients of Anti-Human IgG1, the high micro-channel height, and the slow flow rate are reasons for the limitation of mass transport.
3. The apparent association rate constant *k′_a_* and the apparent dissociation rate constant *k′_d_* of (Human IgG1)-(Anti-Human IgG1) pairs found by the basic kinetic analysis are not the real constants of the specific binding reaction because of these above-mentioned reasons, and needed to be corrected.
4. We propose a modified method to improve the basic kinetic analysis to obtain the calibrated *k_a_* and *k_d_*. Using the calibrated *k_a_* and *k_d_* to simulate reaction curves, we obtain the simulation results more consistent with the experiment results than those using the “apparent” *k′_a_* and *k′_d_*.

## Supplementary Information



## Figures and Tables

**Table 1.** Reaction rate constants of the (Human IgG1)-(Anti-Human IgG1) binding reaction in the PBS solution by the basic kinetic analysis for three supplements of Anti-Human IgG1 solution: (A) 800 μL, (B) 500 μL, and (C) 100 μL. Here *K′_D_* = *k′_d_*/*k′_a_*.

(A)	*k′_a_* (M^−1^ s^−1^)	*k′_d_* (s^−1^)	*K′_D_* (M)
Method 1 Method 2	1.27 × 10^4^ 1.20 × 10^4^	1.60 × 10^−3^ 1.60 × 10^−3^	1.26 × 10^−7^ 1.33 × 10^−7^
(B)	*k′_a_* (M^−1^ s^−1^)	*k′_d_* (s^−1^)	*K′_D_* (M)
Method 1 Method 2	1.58 × 10^4^ 1.69 × 10^4^	2.80 × 10^−3^ 2.90 × 10^−3^	1.77 × 10^−7^ 1.72 × 10^−7^
(C)	*k′_a_* (M^−1^ s^−1^)	*k′_d_* (s^−1^)	*K′_D_* (M)
Method 1 Method 2	1.89 × 10^4^ 1.93 × 10^4^	4.01 × 10^−3^ 4.06 × 10^−3^	2.12 × 10^−7^ 2.10 × 10^−7^

**Table 2.** The *k′_a_* table.

*k_a_*	2 × 10^4^	5 × 10^4^	8 × 10^4^	11 × 10^4^	15 × 10^4^
*k_d_*	0.9 × 10^−4^				
*k′_a_*	4.68 × 10^3^	1.28 × 10^4^	1.87 × 10^4^	2.93 × 10^4^	4.76 × 10^4^
*k_d_*	2 ×10^−4^				
*k′_a_*	4.30 × 10^3^	1.17 × 10^4^	1.67 × 10^4^	2.95 × 10^4^	4.14 × 10^4^
*k_d_*	5 × 10^−4^				
*k′_a_*	4.01 × 10^3^	1.14 × 10^4^	1.48 × 10^4^	2.84 × 10^4^	4.39 × 10^4^
*k_d_*	8 × 10^−4^				
*k′_a_*	4.26 × 10^3^	9.80 × 10^3^	1.38 × 10^4^	2.78 × 10^4^	4.17 × 10^4^
*k_d_*	11 × 10^−4^				
*k′_a_*	4.08 × 10^3^	9.76 × 10^3^	1.41 × 10^4^	2.86 × 10^4^	3.93 × 10^4^
*k_d_*	30 × 10^−4^				
*k′_a_*	3.78 × 10^3^	9.61 × 10^3^	1.33 × 10^4^	2.30 × 10^4^	3.39 × 10^4^
