# Supplementary material for: A Combined Experimental and Theoretical Study on the Immunoassay of Human Immunoglobulin Using a Quartz Crystal Microbalance
Source: Sensors (Basel). 2010 Dec 15;10(12):11498–511. doi: 10.3390/s101211498 (PMC3231060; doi:10.3390/s101211498)

Supplementary Information

Figure 7. The normalized experimental and simulated binding reaction curves for the 500 μL supplement volume. Here the concentration of the Anti-Human IgG1 solution is **(A)** 50 μg/mL, **(B)** 25 μg/mL, **(C)** 10 μg/mL, and **(D)** 5 μg/mL.

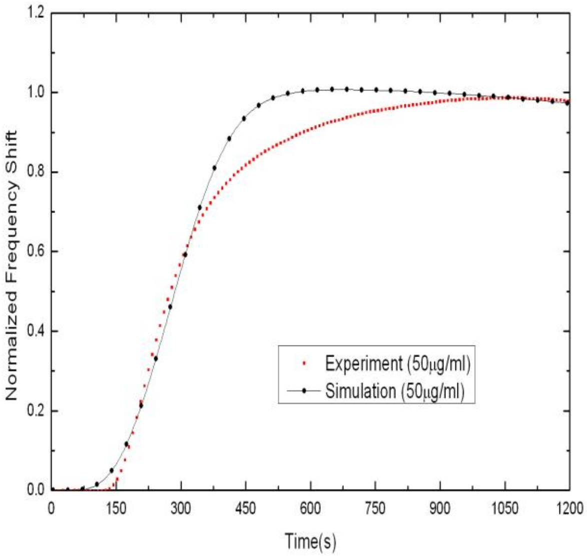

(A)

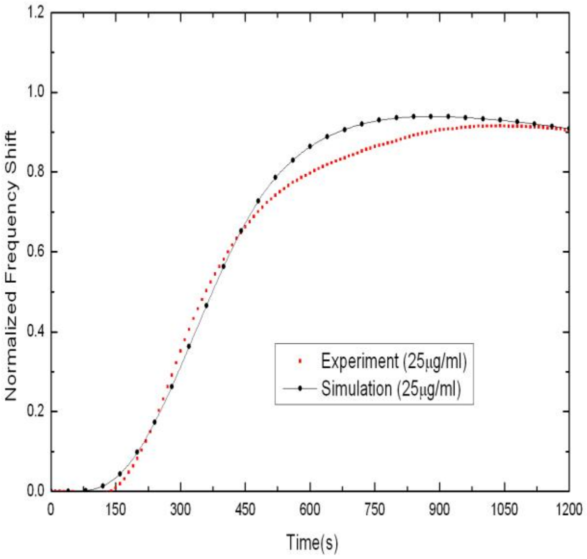

(B)

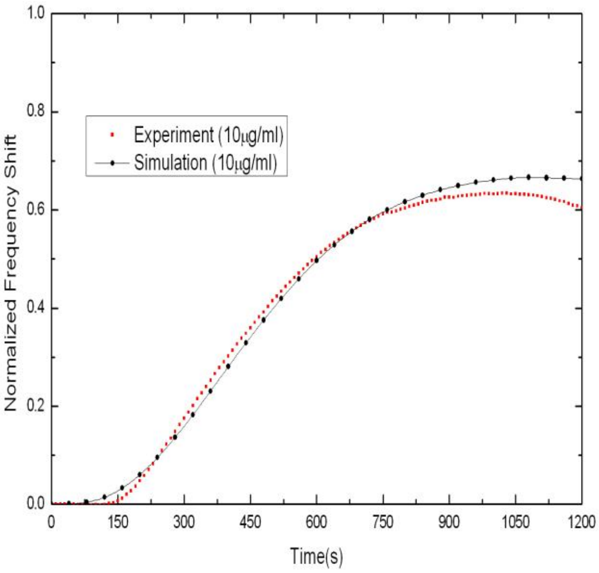

(C)

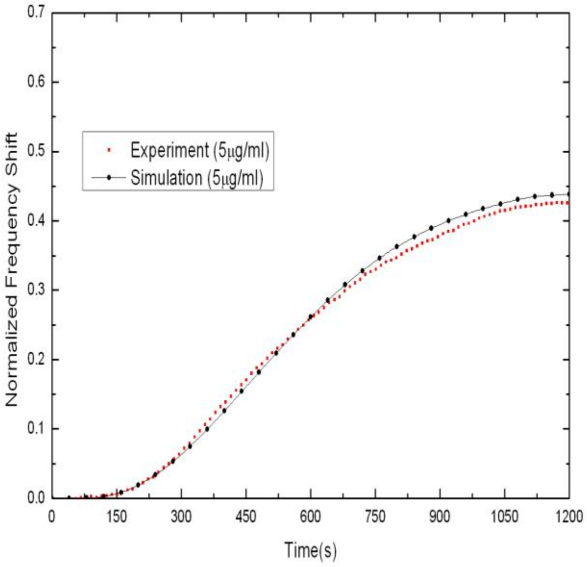

(D)

Figure 8. The normalized experimental and simulated binding reaction curves for the 100 μL supplement volume. Here the concentration of the Anti-Human IgG1 solution is **(A)** 50 μg/mL, **(B)** 25 μg/mL, **(C)** 10 μg/mL, and **(D)** 5 μg/mL.

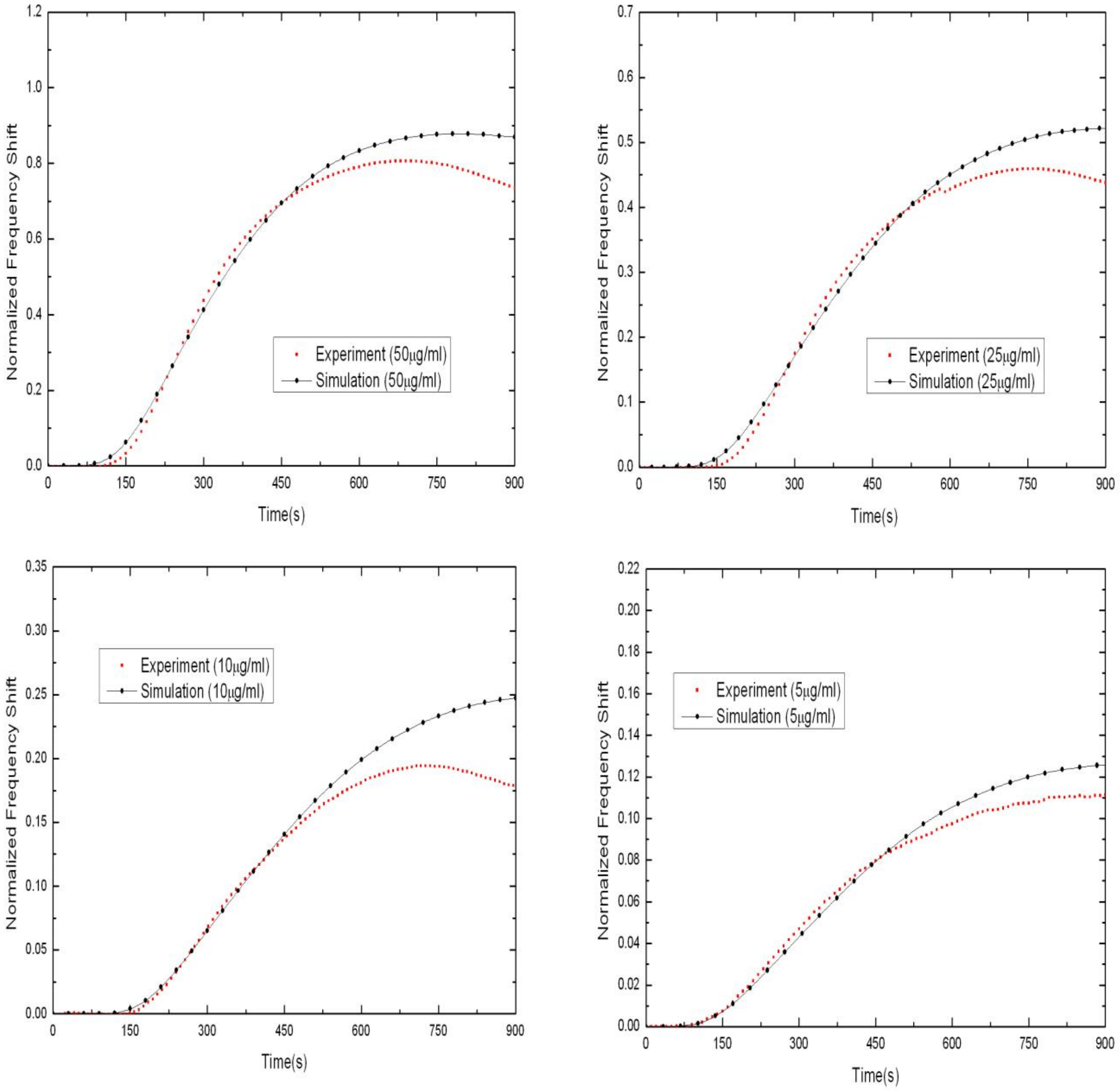

Supplement: Supplementary file 1 [file sensors-10-11498-s001.pdf]
